# The association between relevant comorbidities and dementia in patients with atrial fibrillation

**DOI:** 10.1007/s11357-018-0029-8

**Published:** 2018-06-22

**Authors:** Per Wändell, Axel C. Carlsson, Jan Sundquist, Kristina Sundquist

**Affiliations:** 1 Division of Family Medicine and Primary Care, Department of Neurobiology, Care Sciences and Society, Karolinska Institutet, Huddinge, Sweden; 2 Division of Family Medicine, NVS Department, Karolinska Institutet, Alfred Nobels allé 23, 14183 Huddinge, Sverige; 3 Department of Medical Sciences, Cardiovascular Epidemiology, Uppsala University, Uppsala, Sweden; 4 Center for Primary Health Care Research, Lund University, Malmö, Sweden; 5 Department of Family Medicine and Community Health, Department of Population Health Science and Policy, Icahn School of Medicine at Mount Sinai, New York, USA; 6 Center for Community-based Healthcare Research and Education (CoHRE), Department of Functional Pathology, School of Medicine, Shimane University, Matsue, Japan

**Keywords:** Atrial fibrillation, Dementia, Gender, Hypertension

## Abstract

Risk of dementia is increased in patients with atrial fibrillation (AF). We aimed to study associations between relevant comorbidities and prevalent as well as incident dementia in AF patients. Study population included all adults (*n* = 12,283) ≥ 45 years diagnosed with AF at 75 primary care centers in Sweden 2001–2007. Logistic regression was used to calculate odds ratios (OR) with 95% confidence intervals (95% CI) for associations between comorbidities and prevalent dementia. In a subsample (*n* = 12,096), (excluding patients with dementia diagnosed before AF onset), Cox regression was used to estimate hazard ratios (HR) with 95% CIs for association between comorbidities, and incident dementia, after adjustment for age, socioeconomic factors and anticoagulant treatment. Totally 937 patients (7.6%), 388 men (5.8%) and 549 women (9.7%), were diagnosed with dementia. After adjustments, prevalent dementia was more common in (ORs, 95% CI) men with congestive heart failure (CHF), stroke, and depression; in women with CHF and depression, and among women > 75 years with stroke, but less common in women with hypertension. During a 5.6-year follow-up (standard deviation 2.5), 750 patients (6.2%; 322 men, 4.9%, and 428 women, 7.8%) were diagnosed with incident dementia. An increased risk of incident dementia was found among men with diabetes and depression; a decreased risk among men and women with CHF, and among women with hypertension, myocardial infarction cerebrovascular diseases. The findings regarding incident dementia need to be interpreted with great caution, as they may have been subject to survival bias.

**Electronic supplementary material:**

The online version of this article (10.1007/s11357-018-0029-8) contains supplementary material, which is available to authorized users.

## Introduction

Atrial fibrillation (AF) is the most common arrhythmia in the world and has a prevalence of 2% in Sweden (Forslund et al. 2013). The most important complication among patients with AF is ischemic stroke (Hobbs et al. 2015), with a fivefold higher risk compared to individuals without AF (Wolf et al. 1991).

However, AF is associated with other diseases, mostly cardiovascular, but also dementia (Alonso and Arenas de Larriva 2016). The most important diseases with an increased risk of dementia are coronary heart disease (CHD) (Deckers et al. 2017), atrial fibrillation (Aldrugh et al. 2017; Jacobs et al. 2015; Shah et al. 2016), stroke (Pendlebury and Rothwell 2009), diabetes (Biessels et al. 2014; Zhang et al. 2017), and depression (Bennett and Thomas 2014; Wiberg et al. 2013). Risk assessment tools for incident stroke in AF, i.e., CHADS_2_ CHA_2_DS_2_-VASc scores, have been shown to be predictive of dementia in patients with AF (Graves et al. 2017; Liao et al. 2015). Thus, it is important to study cardiovascular risk factors for the development of dementia in patients with AF. However, many patients with for instance heart failure die before they develop dementia, and a meta-analysis actually found heart disease to be associated with a lower risk of AD (Xu et al. 2015).

There are also gender differences in patients with AF. AF is more common among men (Forslund et al. 2013), and men are being diagnosed with AF on average 5 years earlier than women (Humphries et al. 2001). On the other hand, women with AF exert both a higher risk of stroke as well as of early mortality than men with AF (Michelena et al. 2010).

Dementia could be prevented in AF, provided that the relevant risk factors are known and we believe that many of the comorbidities in patients with AF are preventable. For example, anticoagulant treatment has been shown to decrease the risk of dementia among patients with AF (Friberg and Rosenqvist 2017), and underuse of anticoagulant treatment correlates with dementia among older patients with AF (Viscogliosi et al. 2017).

The aims herein were therefore to study the association between relevant comorbidities and dementia among men and women with AF in Swedish primary care, and to study factors associated with a first hospital diagnosis of dementia among patients with AF who still has not developed dementia.

## Methods

### Design

The study used individual-level patient data from 75 primary health care centers (PHCCs), 48 of which were located in Stockholm County. Individuals attending any of the participating PHCCs between 2001 and 2008 were included in the study. We used *Extractor* software (http://www.slso.sll.se/SLPOtemplates/SLPOPage1____10400.aspx; accessed September 19, 2010) to extract individual electronic patient records (EPRs). National identification numbers were replaced with new unique serial numbers to ensure anonymity. The files were linked to a dataset including data from the Total Population Register, the National Patient Register (NPR), and the Swedish Cause of Death Register, which contains individual-level data on age, sex, education, cause of death, and hospital diagnosis for all residents registered in Sweden. Ethical approvals were obtained from regional boards at Karolinska Institutet and the University of Lund.

### Study population

The study included all patients with diagnosed AF, identified by the presence of the ICD-10 code (10th version of the WHO’s International Classification of Diseases) for atrial fibrillation (I48) in patients’ medical records at the PHCCs. The following cardiovascular-related disorders were used as covariates: hypertension, coronary heart disease (CHD), congestive heart failure (CHF), cerebrovascular diseases (CVD), obesity, diabetes mellitus, depression, and COPD (for specific codes, see Supplementary material). In total, 12,283 individuals (6646 men and 5637 women), aged 45 years or older at the time of AF diagnosis and who visited any of the 75 participating PHCCs from January 1, 2001, until December 31, 2007, with data on neighborhood socioeconomic status available, were included in the study. In the subset studying first hospital dementia diagnosis, patients with an earlier dementia were excluded (*n* = 187), yielding 12,096 patients (6580 men and 5516 women) in the analysis.

### Outcome variable

For logistic regression: prevalent dementia; and for Cox regression: time from first AF diagnosis to first hospital diagnosis of dementia (with the following groups: F00 Alzheimer’s disease, F01 Vascular dementia, F02 Other dementia diseases, F03 Unspecified dementia, F10.7A Alcohol-related dementia, G30 Alzheimer) (until December 31, 2010).

### Demographic and socioeconomic variables

*Sex* was stratified into men and women.

Individuals were divided into the following *age groups* 45–54, 55–64, 65–74, 75–84, and ≥ 85 years. Individuals younger than 45 years were excluded.

*Educational level* was categorized as ≤ 9 years (partial or complete compulsory schooling), 10–12 years (partial or complete secondary schooling), and > 12 years (college and/or university studies), with missing data from 1.041 individuals.

*Marital status* was classified as married, unmarried, divorced, or widowed, with missing data from 50 individuals.

*Neighborhood socioeconomic status (SES)* was categorized into three groups according to the neighborhood index: more than one standard deviation (SD) below the mean (high SES or low deprivation), more than one SD above the mean (low SES or high deprivation), and within one SD of the mean (middle SES or deprivation). The neighborhood index was based derived from the following four variables: low educational status (< 10 years of formal education), low income (< 50% of the median individual income from all sources), unemployment, and receipt of social welfare. The neighborhood deprivation index was categorized into three groups: more than one standard deviation (SD) below the mean (high SES or low deprivation level), more than one SD above the mean (low SES or high deprivation level), and within one SD of the mean (moderate SES or moderate deprivation level).

### Statistical analyses

Analyses were performed stratified by sex. Differences in means and distributions between individuals with or without dementia were compared by Student’s *t* test, chi-square analysis, and Fisher’s exact test. Age-adjustment for comorbidity was performed by logistic regression, and for marital status and socioeconomic factors by ANCOVA.

For individuals with prevalent dementia, i.e., earlier diagnosed, multivariate logistic regression was performed to study the associations with comorbidities adjusting for age and socioeconomic factors (*n* = 11,209, with data missing on marital status in 50 and educational level in 1041 individuals).

For patients with incident dementia, excluding those with a dementia diagnosis before the first registered AF diagnosis (*n* = 187), follow-up analyses were performed by using Cox regression estimating hazard ratios (HRs) with 95% confidence intervals (CIs), using time to the first hospital diagnosis of dementia as the outcome (*n* = 12,096). All models were presented stratified by sex. The regression models were adjusted for the following variables in separate models: for each comorbidity separately adjusted for age and sociodemographic factors (educational level, marital status, and neighborhood socioeconomic status); and a multivariate model also including adjustment for all comorbidities (hypertension, CHD, CHF, stroke, diabetes, obesity, COPD, depression, and anxiety) and anticoagulant treatment.

As supplementary analyses, we also calculated results for newly diagnosed dementia including CHA_2_DS_2_-VASc scores, with incidence rates per 100 patient-years, and trend analysis by Cuzick’s non-parametric trend test, stratified by sex. We also estimated incidence rates of mortality per 100 patient-years for both prevalent and incident dementia, and for all comorbidities, also using HRs by Cox regression in fully adjusted models. Cox regression analysis was also performed in fully adjusted models with exclusion of patients without dementia and who died during follow-up, as a sensitivity analysis.

A *p* value for two-sided tests of < 0.01 was considered statistically significant in baseline comparisons due to the multiple comparisons between men and women. A two-sided *p* value of < 0.05 was considered statistically significant for variables in the logistic regression and Cox regression. All analyses were performed in STATA 15.2.

## Results

Characteristics of the entire study population consisting of patients with AF (*n* = 12,283), stratified by sex (6646 men and 5637 women), and into those with a diagnosis of dementia (yes/no) are shown in Table 1.

**Table 1:** Baseline characteristics for patients aged ≥ 45 years with diagnoses of AF (*N* = 12,283), and with or without dementia in primary care attending the 75 PHCCs between January 1, 2001 and December 31, 2007

	Men (*N* = 6646)			Women (*N* = 5637)		
	No dementia	Dementia	Difference	No dementia	Dementia	Difference
	*N* = 6258	*N* = 388	Age-adjusted	*N* = 5088	*N* = 549	Age-adjusted
	Numbers (%)	Numbers (%)	*p* values	Numbers (%)	Numbers (%)	*p* values
Number of deaths	1723 (27.5)	260 (67.0)	< 0.001	1631 (32.1)	340 (61.9)	< 0.001
Age (years), mean (SD)	71.7 (10.2)	78.9 (6.8)	< 0.001	76.6 (9.4)	82.0 (6.2)	< 0.001
Age groups (years)			< 0.001			< 0.001
45–54	369 (5.9)	1 (0.3)		105 (2.1)	0 (0.0)	
55–64	1209 (19.3)	13 (3.4)		513 (10.1)	8 (1.5)	
65–74	1974 (31.5)	68 (17.5)		1222 (24.0)	44 (8.0)	
75–79	1143 (18.3)	114 (29.4)		1062 (20.9)	108 (19.7)	
80–84	975 (15.6)	108 (27.8)		1169 (23.0)	195 (35.5)	
≥ 85	588 (9.4)	84 (21.7)		1017 (20.0)	194 (35.3)	
Educational level			0.70			0.10
Compulsory schooling	2335 (39.3)	151 (42.9)		2336 (52.0)	263 (57.8)	
Secondary schooling	2241 (37.7)	126 (35.8)		1493 (33.2)	135 (29.7)	
College and/or university studies	1362 (22.9)	75 (21.3)		667 (14.8)	57 (12.5)	
Marital status			0.99			0.43
Married	3719 (59.7)	231 (59.7)		1550 (30.6)	113 (20.7)	
Unmarried	613 (9.8)	17 (4.4)		366 (7.2)	33 (6.1)	
Divorced	974 (15.6)	47 (12.1)		730 (14.4)	62 (11.4)	
Widowed	928 (14.9)	92 (23.8)		2420 (47.8)	337 (61.8)	
Neighborhood SES			0.55			0.36
High	2487 (39.7)	169 (43.6)		1734 (34.1)	214 (39.0)	
Middle	2860 (45.7)	170 (43.8)		2505 (49.2)	272 (49.5)	
Low	911 (14.6)	49 (12.6)		849 (16.7)	63 (11.5)	
Diagnosis						
Hypertension	2577 (41.2)	149 (38.4)	0.53	2504 (49.2)	219 (39.9)	< 0.001
Coronary heart disease	1594 (25.5)	128 (33.0)	0.23	1357 (26.7)	155 (28.2)	0.28
Congestive heart failure	2637 (42.1)	219 (56.4)	0.042	2476 (48.7)	352 (64.1)	0.001
Cerebrovascular diseases	1149 (18.4)	128 (33.0)	< 0.001	1137 (22.4)	152 (27.7)	0.21
Obesity	346 (5.5)	6 (1.6)	0.14	253 (5.0)	9 (1.6)	0.11
Diabetes mellitus	1653 (26.4)	109 (28.1)	0.096	1799 (33.4)	188 (34.2)	0.54
COPD	677 (10.8)	33 (8.5)	0.049	637 (12.5)	69 (12.6)	0.40
Depression	369 (5.9)	43 (11.1)	< 0.001	550 (10.8)	77 (14.0)	0.009
Anxiety	168 (2.7)	15 (3.9)	0.23	273 (5.4)	40 (7.3)	0.033

Information on educational level and marital status is missing for some individuals

Odds from the multivariate logistic regression models of the association with prevalent dementia are shown in Table 2, stratified by sex, and also stratified by age group, i.e., 45–74 years and ≥ 75 years. Dementia was significantly less common among women with hypertension in all ages combined and in women ≥ 75 years. Dementia was consistently more common among all men and women with heart failure, and stratified by age group among women ≥ 75 years. Furthermore, dementia was more common among men with cerebrovascular diseases, also in both age groups, but among women only among those 45–74 years of age. Dementia was also more common in men with depression, also in both age groups, but among women only when combining all ages. When looking at men and women combined, the fully adjusted ORs with 95% CI (with interaction term between sex and cerebrovascular diseases) were for hypertension 0.81 (0.69–0.94), heart failure 1.35 (1.15–1.58), cerebrovascular diseases 1.83 (1.44–2.31), obesity 0.55 (0.32–0.95), and depression 1.59 (1.27–2.00).

**Table 2:** Multivariate logistic regression for the association between dementia and comorbidities for patients aged ≥ 45 years with diagnoses of AF (*n* = 11,209) in primary care attending the 75 PHCCs between January 1, 2001 and December 31, 2007

Number of patients	Men			Women		
	All	< 75 years	≥ 75 years	All	< 75 years	≥ 75 years
	*N* = 6273	*N* = 3578	*N* = 2695	*N* = 4936	*N* = 1842	*N* = 3094
	OR (95% CI)	OR (95% CI)	OR (95% CI)	OR (95% CI)	OR (95% CI)	OR (95% CI)
Diagnosis						
Hypertension	0.91 (0.72–1.14)	0.90 (0.57–1.44)	0.89 (0.68–1.15)	*0.73 (0.60–0.90)*	0.64 (0.35–1.16)	*0.74 (0.60–0.92)*
Coronary heart disease	1.13 (0.89–1.45)	1.16 (0.69–1.93)	1.09 (0.83–1.43)	0.87 (0.70–1.10)	0.67 (0.31–1.46)	0.89 (0.70–1.13)
Congestive heart failure	*1.27 (1.01–1.61)*	1.49 (0.93–2.37)	1.20 (0.92–1.57)	*1.43 (1.15–1.76)*	1.30 (0.71–2.39)	*1.40 (1.12–1.76)*
Cerebrovascular diseases	*1.82 (1.43–2.31)*	*2.76 (1.71–4.44)*	*1.56 (1.18–2.05)*	1.11 (0.89–1.39)	*2.08 (1.08–4.01)*	1.04 (0.82–1.32)
Obesity	0.54 (0.23–1.25)	0.14 (0.02–1.06)	1.02 (0.40–2.63)	0.54 (0.26–1.12)	0.62 (0.18–2.12)	0.48 (0.19–1.20)
Diabetes mellitus	1.18 (0.92–1.52)	1.40 (0.87–2.27)	1.07 (0.80–1.44)	1.05 (0.85–1.29)	1.15 (0.63–2.10)	1.05 (0.84–1.31)
COPD	0.68 (0.47–1.00)	0.58 (0.23–1.47)	0.71 (0.47–1.09)	1.09 (0.81–1.46)	1.81 (0.87–3.74)	0.98 (0.70–1.35)
Depression	*2.04 (1.41–2.94)*	*2.81 (1.48–5.37)*	*1.72 (1.10–2.68)*	*1.38 (1.03–1.85)*	1.78 (0.82–3.86)	1.33 (0.97–1.83)
Anxiety	1.33 (0.72–2.42)	2.02 (0.69–5.89)	1.21 (0.59–2.48)	1.33 (0.88–1.99)	1.64 (0.58–4.61)	1.26 (0.81–1.96)

Models are adjusted for age, socioeconomic factors, and all comorbidities. Information on educational level and marital status is missing for some individuals. Italicized values are statistically significant

In the analysis of incident dementia, i.e., a first hospital diagnosis of dementia, excluding patients with a recorded earlier dementia (*n* = 187), the study sample consisted of 12,096 individuals (6580 men and 5516 women). In total, 750 patients (6.2%) were diagnosed with dementia during the follow-up of whom 322 were men (4.9%) and 428 were women (7.8%). The mean follow-up time until first hospital diagnosis of dementia in those without previous dementia was 5.6 years (standard deviation 2.5). Patients were followed for a total of 69,214 person-years: 38,184 person-years for men and 31,030 person-years for women.

The risks of a first hospital diagnosis for dementia are shown in Table 3. For men, diabetes and depression were significantly associated with an increased risk of incident dementia, and CHF with a decreased risk. For women, hypertension, myocardial infarction, CHF, and stroke significantly associated with a decreased risk of incident dementia. We also performed analyses with exclusion of patients without dementia who died during follow-up (Supplementary Table 1). In fully adjusted models, only an increased risk in patients with anxiety and decreased risk of anticoagulant treatment among women were statistically significant.

**Table 3:** Cox regression models (with hazard ratios (HRs) and 95% confidence interval (CI)) for incident hospital diagnosis of dementia among patients aged ≥ 45 years with diagnoses of AF (*n* = 11,063) in primary care attending the 75 PHCCs between January 1, 2001 and December 31, 2007; patients with an earlier known hospital episode of dementia before AF diagnosis excluded

	Men (*n* = 6216)		Women (*n* = 4847)	
	Model 1	Model 2	Model 1	Model 2
Diagnosis				
Hypertension	0.98 (0.78–1.24)	0.95 (0.75–1.21)	*0.78 (0.63–0.96)*	*0.77 (0.62–0.95)*
Myocardial infarction	0.87 (0.61–1.24)	0.92 (0.64–1.33)	*0.51 (0.34–0.77)*	*0.52 (0.35–0.79)*
Congestive heart failure	*0.71 (0.55–0.90)*	*0.71 (0.56–0.91)*	*0.65 (0.53–0.81)*	*0.68 (0.55–0.85)*
Cerebrovascular diseases	1.02 (0.77–1.36)	0.98 (0.73–1.31)	*0.75 (0.57–0.98)*	*0.69 (0.52–0.91)*
Ischemic stroke	1.08 (0.81–1.45)	–	*0.75 (0.57–0.99)*	–
Bleeding stroke	0.60 (0.22–1.62)	–	0.75 (0.28–2.02)	–
Obesity	0.58 (0.24–1.43)	0.54 (0.22–1.32)	0.64 (0.30–1.35)	0.62 (0.29–1.34)
Diabetes mellitus	*1.40 (1.08–1.83)*	*1.50 (1.15–1.96)*	1.07 (0.83–1.39)	1.20 (0.92–1.56)
COPD	0.77 (0.52–1.15)	0.80 (0.54–1.20)	1.24 (0.93–1.67)	1.28 (0.95–1.72)
Depression	*1.67 (1.14–2.44)*	*1.54 (1.04–2.28)*	1.18 (0.87–1.61)	1.16 (0.85–1.59)
Anxiety	1.56 (0.87–2.78)	1.46 (0.80–2.65)	1.24 (0.82–1.88)	1.23 (0.80–1.88)
Anticoagulant treatment	–	*0.78 (0.61–0.98)*	–	*0.81 (0.65–0.99)*

Model 1 is for each comorbidity separately and adjusted for age and sociodemographic variables (educational level, marital status, and neighborhood socioeconomic status). Model 2 is multivariate with adjustment as in model 1 but also for all comorbidities (hypertension, myocardial infarction, congestive heart failure, cerebrovascular diseases, obesity, diabetes, COPD, depression, and anxiety) and anticoagulant treatment. Significant interaction was found between age and marital status. Italicized values are statistically significant

Results were also estimated for a first hospital diagnosis of dementia by CHA_2_DS_2_-VASc score, showing a statistically significant trend for men (Supplementary Table 2). The lowest scores associated with an incidence rate of 0.5 per 100 patient-years and above were for men two, and for women three. An incidence rate of 1.0 and above for men and women was seen for scores of three and above.

We also estimated the mortality rates for the different comorbidities, as well as a fully adjusted Cox regression model with time to death as outcome (Supplementary Table 3). Among men with a specific comorbidity vs without this comorbidity, higher mortality rates as well as significantly higher HRs were found for myocardial infarction, CHF, stroke, COPD, and depression, and for women for myocardial infarction, CHF, stroke, diabetes and COPD, and a lower incidence rate and lower HR for hypertension. We also estimated mean age for new diagnoses and for mean age at death for dementia and CHF. Mean age for patients with a new dementia diagnosis was among men 83.0 years, and among women 85.2 years. Mean age for patients with a new diagnosis of CHF was among men 79.9 years and among women 83.3 years. Mean age at death for patients with a new dementia diagnosis was among men 83.6 years and for women 86.6 years. Mean age at death for patients with a new diagnosis of CHF was among men 80.3 years and among women 83.8 years.

## Discussion

The main finding of this study was the different risk factor patterns between men and women for incident dementia, defined as a first hospital diagnosis of dementia. For men, diabetes and depression were risk factors for incident dementia, while for women, hypertension and myocardial infarction was associated with a lower risk. Besides, CHF was associated with a lower risk in men and women, and stroke with a lower risk among women.

Some of the associations could be expected, while others were more surprising. A lower risk associated with CHF in men and women, and also with myocardial infarction and stroke in women, could be due to the higher mortality in these patients, and thus a higher risk that they die before the development of dementia. The lower risk of dementia in patients with stroke is puzzling. One possibility is that patients with stroke will not be further examined for dementia, or the risk of dementia might also be overlooked. Furthermore, as dementia is a disorder often developing slowly over many years, the diagnosis could be set rather late in the course of the disease. In the sensitivity analyses, the lower risk estimates for MI, CHF, and stroke were attenuated and no longer significant, supporting our interpretation. An earlier meta-analysis actually found heart disease to be associated with a lower risk of AD (Xu et al. 2015). However, all these factors raise the question of the possibility to study incident dementia within this setting. Diseases such as myocardial infarction or stroke develop fast, which enables register-studies, while other diseases such as dementia often develop slowly and are diagnosed later in the course, which could produce misleading results.

We also performed analyses using logistic regression, in contrast to the longitudinal analyses of the Cox regression, and finding different results regards some of the background diagnoses, i.e., both CHF and cerebrovascular diseases was associated with a higher risk of dementia, at least in some subgroups. Some of the findings in the logistic regression and Cox regression were consistent, i.e., the lower dementia risk in women with hypertension and the higher risk in men with depression. The lack of consistence in the analyses for CHF and cerebrovascular diseases in the association with dementia could thus suggest that the findings for incident dementia in the Cox regression could be due to bias, such as survival bias (Delgado-Rodriguez and Llorca 2004).

Diabetes is known to be associated with dementia, but we only found this association statistically significant among men. As men are diagnosed earlier than women with diabetes, the lower age at onset and consequently a longer risk exposition could be one explanation to our findings (Wandell and Carlsson 2014), while women with diabetes might have a higher risk to die before they develop dementia. Another explanation could be that women have a longer life expectancy than men, and thus have a higher risk of developing dementia, with competing factors other than diabetes being more prominent. For depression, women more often develop this mental disorder, but possibly men with depression could be affected to a larger degree. We have earlier found depression to be associated with increased mortality among men with AF, but not among women (Wandell et al. 2016).

Regarding the lower risk among women with hypertension, this could possibly be due to a positive effect by antihypertensive drugs, even if our findings should be interpreted with caution. However, this finding is in line with conclusions in a review from The Swedish Council on Health Technology Assessment in Health Care (SBU), i.e., that a good control of blood pressure in middle age reduces the risk of dementia, and also that antihypertensive drug treatment reduces the risk of developing stroke-related dementia (SBU, 2008). However, as regards specific antihypertensive treatment, reviews and meta-analyses have shown somewhat different results, with one review stating, that “antihypertensive drugs, particularly calcium channel blockers and renin–angiotensin system blockers, may be beneficial in preventing cognitive decline and dementia”, but also that “randomized controlled trials and meta-analyses have sometimes produced conflicting results, but these are probably due to methodological considerations”, (Rouch et al. 2015).

We also found CHA_2_DS_2_-VASc to be related to incident dementia among men, in line with earlier findings (Graves et al. 2017; Liao et al. 2015). Anticoagulant (AC) treatment is shown to decrease the risk of dementia among AF patients (Friberg and Rosenqvist 2017), and underuse of AC correlates with dementia among older patients with AF (Viscogliosi et al. 2017). Thus, as a CHA_2_DS_2_-VASc score of two among men and three among women is regarded as an indication for anticoagulant treatment, and this seems to be a useful cut-off also for dementia prevention.

There are several limitations of this study. The study sample is a subgroup of the AF population, i.e., patients with concomitant diagnoses of AF and dementia registered in primary health care. Results cannot be generalized to all AF or dementia patients or to patients in other settings. The findings may have been subject to survival bias (Delgado-Rodriguez and Llorca 2004). Besides, it could be possible that patients with an earlier stroke are not examined for dementia, or even that the risk of dementia is overlooked. All these mentioned factors could have affected the results, and yielded discrepant findings. Moreover, AF could not be classified as paroxysmal, persistent or permanent and heart rhythm could not be classified as sinus rhythm or fibrillation rhythm.

A major strength of this study was that we were able to link clinical data from individual EPRs to data from national demographic and socioeconomic registers with less than 1% of information missing. While many previous follow-up studies of AF have used hospital data, the current study used data from primary care, which may better reflect the risks associated with AF in less severe cases.

In conclusion, in this clinical setting with patients with AF treated in primary care, we found incident dementia was positively associated with diabetes and depression to among men, and negatively with hypertension among women. Further studies in patients with AF from a primary care setting with data on severity of dementia, and of use of antihypertensive agents in relation to incident dementia are needed to further understand our findings. Besides, studies exploring the lack of association between stroke and dementia are needed, to see whether stroke patients are examined for possible dementia and are registered with this diagnosis if accurate.

## Electronic supplementary material

ESM 1 (DOCX 20 kb)
