# Supplementary material for: The association between relevant comorbidities and dementia in patients with atrial fibrillation
Source: GeroScience. 2018 Jun 22;40(3):317–24. doi: 10.1007/s11357-018-0029-8 (PMC6060202; doi:10.1007/s11357-018-0029-8)
Supplement: Supplementary file 1 — (DOCX 20 kb) [file 11357_2018_29_MOESM1_ESM.docx]

**Supplementary material**

*Co-morbidities with ICD-10 codes*

We identified the following cardiovascular co-morbidities from the EPRs among the individuals in the study population: hypertension (I10-15); coronary heart disease (CHD; I20-25), also including registered hospitalizations for myocardial infarction from the NPR; congestive heart failure (CHF; I50 or I110), also including hospitalizations for CHF from the NPR; cerebrovascular diseases (CVD; I60-69), also including registered hospitalizations for ischemic or haemorrhagic stroke from the NPR; diabetes mellitus (E10-14); obesity (E65-E68); COPD (J40-J47); depression (F32–F34, F38–F39); and anxiety disorders (F40–41).

**Supplementary Table 1**

Cox regression models (with hazard ratios (HRs) and 95% confidence interval (CI)) for incident hospital diagnosis of dementia among patients aged ≥45 years with diagnoses of AF (*n*=8,335) in primary care attending the 75 PHCCs between January 1^st^ 2001 and December 31^st^ 2007; patients with an earlier known hospital episode of dementia before AF diagnosis excluded, as well as people without dementia who died during follow-up

|  | Men (n=4,712) |  | Women (n=3,623) |
| --- | --- | --- | --- |
| Diagnosis |  |  |  |
| Hypertension | 1.04 (0.71-1.85) |  | 0.90 (0.56-1.45) |
| Myocardial infarction | 1.13 (0.99-1.29) |  | 0.92 (0.60-1.12) |
| Congestive heart failure | 1.21 (0.83-1.76) |  | 0.91 (0.73-1.14) |
| Cerebrovascular diseases | 0.95 (0.70-1.28) |  | 0.94 (0.71-1.24) |
| Ischemic stroke |  |  |  |
| Bleeding stroke |  |  |  |
| Obesity | 0.73 (0.29-1.86) |  | 0.90 (0.41-1.95) |
| Diabetes mellitus | 1.17 (0.89-1.55) |  | 1.07 (0.81-1.40) |
| COPD | 0.84 (0.56-1.26) |  | 1.34 (0.99-1.82) |
| Depression | 1.29 (0.86-1.95) |  | 1.06 (0.76-1.48) |
| Anxiety | 1.08 (0.58-1.99) |  | **1.59 (1.02-2.47)** |
| Anticoagulant treatment | 0.88 (0.69-1.11) |  | **0.76 (0.61-0.94)** |

Models adjusted for age and socio-demographic variables (educational level, marital status and neighborhood socio-economic status), co-morbidities (hypertension, myocardial infarction, congestive heart failure, cerebrovascular diseases, obesity, diabetes, COPD, depression and anxiety) and anticoagulant treatment, and with people without dementia and who died during follow-up excluded (significant interaction between neighborhood socio-economic status and age for men)

**Supplementary Table 2**.

CHA_2_DS_2_-VASc scores and incidence of dementia for patients with AF, stratified by sex.

| CHA_2_DS_2_-VASc | Men | | | Women | | |
| --- | --- | --- | --- | --- | --- | --- |
| score | No dementia | Dementia | | No dementia | Dementia | |
|  | n | n (%) | Incidence rate | N | n (%) | Incidence rate |
| 0 | 337 | 2 (0.6) | 0.10 (0.03-0.41) | - | - | - |
| 1 | 721 | 6 (0.8) | 0.14 (0.06-0.30) | 109 | 0 (0.0) | 0.00 |
| 2 | 1,478 | 69 (4.5) | 0.75 (0.59-0.95) | 279 | 6 (2.1) | 0.36 (0.16-0.80) |
| 3 | 1,755 | 116 (6.2) | 1.08 (0.90-1.30) | 916 | 100 (9.8) | 1.72 (1.41-2.09) |
| 4 | 1,284 | 90 (6.6) | 1.14 (0.93-1.41) | 1,642 | 156 (8.7) | 1.54 (1.32-1.80) |
| 5 | 549 | 28 (4.9) | 0.85 (0.58-1.23) | 1,391 | 116 (7.7) | 1.40 (1.17-1.68) |
| 6 | 130 | 11 (7.8) | 1.44 (0.80-2.60) | 619 | 40 (6.1) | 1.08 (0.79-2.41) |
| 7 | 4 | 0 (0.0) | 0.00 | 123 | 9 (6.8) | 1.25 (0.65-5.18) |
| 8 | 0 | 0 (0.0) | NA | 9 | 1 (10.0) | 1.92 (0.27-13.65) |
| 9 | - | - | - | 0 | 0 (0.0) | NA |
| All | 6,258 | 322 (4.9) | 0.84 (0.76-0.94) | 5,088 | 428 (7.8) | 1.38 (1.25-1.52) |

Incidence rates for dementia with 95% confidence intervals are shown per 100 patient-years at risk

Trend analysis by Cuzick’s non-parametric trend test, stratified by sex, was significant for men (p<0.001) but not for women (p=0.67)

**Supplementary Table 3.**

Mortality incidence rates (per 100 patient-years) and 95% confidence interval (CI)) among patients aged ≥45 years with diagnoses of AF (*n*=11,063) and with or without different co-morbidities in primary care

|  | Men (n=6,216) | | Women (n=4,847) | |
| --- | --- | --- | --- | --- |
|  | Incidence rate (95% CI) | HR (95% CI) | Incidence rate (95% CI) | HR (95% CI) |
|  |  |  |  |  |
| Hypertension | 4.61 (4.29-4.96) | 0.93 (0.84-1.02) | 5.05 (4.71-5.42) | **0.83 (0.75-0.93)** |
|  | 5.22 (4.93-5.52) | 1 (ref) | 6.63 (6.25-7.04) | 1 (ref) |
| Myocardial infarction | 8.40 (7.58-9.30) | **1.46 (1.29-1.66)** | 9.18 (8.23-10.25) | **1.39 (1.20-1.61)** |
|  | 4.53 (4.31-4.76) | 1 (ref) | 5.46 (5.19-5.73) | 1 (ref) |
| Congestive heart failure | 7.98 (7.53-8.46) | **1.59 (1.44-1.76)** | 8.65 (8.16-9.16) | **1.43 (1.29-1.60)** |
|  | 3.25 (3.03-3.48) | 1 (ref) | 3.82 (3.55-4.11) | 1 (ref) |
| Cerebrovascular diseases | 7.19 (6.57-7.88) | **1.42 (1.26-1.59)** | 8.38 (7.70-9.13) | **1.54 (1.36-1.73)** |
|  | 4.52 (4.30-4.76) | 1 (ref) | 5.23 (4.96-5.52) | 1 (ref) |
| Obesity | 2.39 (1.82-3.14) | 0.80 (0.60-1.07) | 2.75 (2.05-3.70) | 0.95 (0.69-1.30) |
|  | 5.12 (4.89-5.35) | 1 (ref) | 6.02 (5.75-6.30) | 1 (ref) |
| Diabetes mellitus | 5.31 (4.82-5.84) | 1.11 (0.99-1.25) | 6.07 (5.49-6.71) | **1.16 (1.02-1.31)** |
|  | 4.88 (4.64-5.13) | 1 (ref) | 5.80 (5.52-6.11) | 1 (ref) |
| COPD | 7.35 (6.55-8.23) | **1.31 (1.14-1.49)** | 7.06 (6.28-7.93) | **1.38 (1.20-1.59)** |
|  | 4.69 (4.47-4.93) | 1 (ref) | 5.69 (5.42-5.97) | 1 (ref) |
| Depression | 6.43 (5.49-7.54) | **1.24 (1.04-1.48)** | 5.76 (5.02-6.60) | 0.99 (0.84-1.17) |
|  | 4.87 (4.65-5.10) | 1 (ref) | 5.87 (5.60-6.16) | 1 (ref) |
| Anxiety | 5.67 (4.41-7.29) | 0.95 (0.72-1.25) | 5.25 (4.29-6.42) | 0.94 (0.74-1.19) |
|  | 4.95 (4.73-5.18) | 1 (ref) | 5.90 (5.63-6.18) | 1 (ref) |
| All dementia | 12.20 (10.80-13.78) | **1.92 (1.66-2.12)** | 11.62 (10.45-12.93) | **1.73 (1.51-1.98)** |
|  | 4.65 (4.44-4.88) | 1 (ref) | 5.52 (5.26-5.80) | 1 (ref) |
| Incident dementia | 10.99 (9.60-12.58) | **1.69 (1.45-1.97)** | 9.76 (8.62-11.06) | **1.47 (1.26-1.71)** |
|  | 4.65 (4.44-4.88) | 1 (ref) | 5.52 (5.26-5.80) | 1 (ref) |

Model 1 is for each co-morbidity separately and adjusted for age and socio-demographic variables (educational level, marital status and neighborhood socio-economic status), Model 2 is multivariate with adjustment as in Model 1 but also for all co-morbidities (hypertension, myocardial infarction, congestive heart failure, cerebrovascular diseases, obesity, diabetes, COPD, depression and anxiety) and anticoagulant treatment. Significant interaction was found between age and marital status.

Bold values are statistically significant.
